# Cancer immunotherapy: how low-level ionizing radiation can play a key role

**DOI:** 10.1007/s00262-017-1993-z

**Published:** 2017-03-30

**Authors:** Marek K. Janiak, Marta Wincenciak, Aneta Cheda, Ewa M. Nowosielska, Edward J. Calabrese

**Affiliations:** 1 Department of Radiobiology and Radiation Protection, Military Institute of Hygiene and Epidemiology, 4 Kozielska St., 01-163 Warsaw, Poland; 2 Department of Environmental Health Sciences, School of Public Health and Health Sciences, Morrill I, N344, University of Massachusetts, Amherst, MA 01003 USA

**Keywords:** Low-level radiation, Carcinogenesis, Immune suppression, Radio-immunotherapy

## Abstract

The cancer immunoediting hypothesis assumes that the immune system guards the host against the incipient cancer, but also “edits” the immunogenicity of surviving neoplastic cells and supports remodeling of tumor microenvironment towards an immunosuppressive and pro-neoplastic state. Local irradiation of tumors during standard radiotherapy, by killing neoplastic cells and generating inflammation, stimulates anti-cancer immunity and/or partially reverses cancer-promoting immunosuppression. These effects are induced by moderate (0.1–2.0 Gy) or high (>2 Gy) doses of ionizing radiation which can also harm normal tissues, impede immune functions, and increase the risk of secondary neoplasms. In contrast, such complications do not occur with exposures to low doses (≤0.1 Gy for acute irradiation or ≤0.1 mGy/min dose rate for chronic exposures) of low-LET ionizing radiation. Furthermore, considerable evidence indicates that such low-level radiation (LLR) exposures retard the development of neoplasms in humans and experimental animals. Here, we review immunosuppressive mechanisms induced by growing tumors as well as immunomodulatory effects of LLR evidently or likely associated with cancer-inhibiting outcomes of such exposures. We also offer suggestions how LLR may restore and/or stimulate effective anti-tumor immunity during the more advanced stages of carcinogenesis. We postulate that, based on epidemiological and experimental data amassed over the last few decades, whole- or half-body irradiations with LLR should be systematically examined for its potential to be a viable immunotherapeutic treatment option for patients with systemic cancer.

**Electronic supplementary material:**

The online version of this article (doi:10.1007/s00262-017-1993-z) contains supplementary material, which is available to authorized users.

## Introduction

The immune system is a crucial player in the organism’s control over the development of neoplasms (reviewed in [1]). After years of controversies, the early concept of cancer immunological surveillance [2, 3], whereby specifically stimulated (adaptive) immunity wards off proliferation of neoplastically transformed cells, has now been incorporated into the modern cancer immunoediting process. During the three phases of this process, the anti-neoplastic immune functions and immunogenicity of cancer cells are being gradually “edited”, so that the immune system protects the host against the development of a malignancy during the initial “elimination” phase, but later, during the following “equilibrium” and, especially, “escape” phases, morphs into an active supporter of cancer progression. Consequently, the emerging tumor not only evades immune recognition and destruction, but also actively contributes to remodeling of its microenvironment towards the immunosuppressive and pro-neoplastic state [4–10].

The improved understanding of the relationship between a growing tumor and the immune system has shed new light on the recently acknowledged complex interactions of ionizing radiation (IR) with cancer-related immunity. This, in turn, has led to the development of novel radiotherapeutic schemes based on the notion that local exposures at moderate (between 0.1 and 2.0 Gy absorbed during acute exposures) or even high doses (over 2.0 Gy) of radiation can, especially in combination with standard immunotherapy, stimulate various anti-neoplastic immune reactions, and/or reverse their suppressive state. These effects are thought to result from the radiation-induced immunogenic types of cell death, local inflammation, and tissue injury, all leading to the emergence of “danger signals” which prompt activities of the non-specific (innate) immune system; extensive recapitulation of the immunomodulatory effects of local radiotherapy (RT) has recently been summarized in a number of excellent reviews [11–20]. However, even moderate radiotherapeutic doses are potentially harmful to the surrounding normal tissues, which can cause immunosuppression and/or induce secondary cancers [21–23]. Such complications are highly unlikely after exposures to low doses (≤0.1 Gy absorbed within a short time or ≤0.1 mGy/min dose rate applied during a protracted exposure) of low linear energy transfer (LET) IR, referred to in this paper as low-level radiation (LLR). Indeed, the effects of exposures to LLR, including modulation of the immune functions, can qualitatively and quantitatively differ from those induced by moderate-to-high doses of low-LET radiation [24–29].

The present paper indentifies and evaluates epidemiological as well as animal studies which indicate that exposures to LLR can inhibit or retard the development of primary and metastatic cancers [27, 30–91]. This evaluation will include an assessment of possible mechanisms by which such protective effects may be mediated including: LLR-induced scavenging of reactive chemical intermediates, stimulation of the repair of the DNA damage, mitigation of inflammation, triggering of selective apoptosis or senescence of aberrant cells, and the up-regulation of both the innate and adaptive arms of the anti-cancer immune system [25, 92–95]. Since enhancing anti-neoplastic immunity may be an important mechanism of the cancer-inhibitory effects of LLR [93–101], clinical trials of whole- or half-body irradiations (WBI or HBI) with LLR are also evaluated [102–106].

This paper will also assess how LLR can affect and modify advanced phases of cancer development resulting in a reversal of suppressed immune functions and/or restoration of the susceptibility of cancer cells to the assaults by immune effectors. However, in contrast to the extensively reviewed relations between moderate- and high-dose RT and the response of the immune system, recapitulations of the similar effects of LLR in the context of their clinical exploitation are virtually nonexistent. The present paper will complement and extend a recent review of the vast pre-clinical evidence of the LLR-induced protective/adaptive response in normal but not neoplastic tissues, which provides arguments for the trials of the LLR-based therapy of cancer [29].

## Immunosuppressive tumor microenvironment

The concept that in vertebrates, elements of the immune system specifically recognize and eliminate incipient neoplastic cells and protect thereby against the development of overt malignancy dates back to late 1950s [2, 3]. In accordance with this “cancer immunosurveillance” hypothesis, it was demonstrated that both immuno-compromised human patients and experimental animals are at increased risk of developing various neoplasms (reviewed in [107]). However, investigations by Stutman showed that chemically induced sarcomas or adenomas do not develop more often in athymic, T-cell-deficient, nude mice than in their wide-type, immunocompetent counterparts [108]. This observation seriously challenged the cancer immunosurveillance model and almost led to its abandonment [7]. Yet, evidence accumulated in recent years has helped to explain what was wrong with the original cancer immunosurveillance hypothesis and why some neoplasms progress to their clinical stage. Thus, it was found that innate immunity initially senses the presence of transformed cells and exercises the first line of anti-cancer defense. Soon after the activation, elements of the innate immune system promote induction of adaptive (specific) anti-tumor responses. However, owing to genetic and epigenetic changes in the developing neoplastic cells, tumors may become “invisible” to immune effectors through loss or aberrant expression of the MHC class I antigens (reviewed in [109, 110]) or of other molecules on cancer cells involved in triggering of the innate and/or adaptive immune responses [111, 112]. For example, a change in hydrophobicity of tumor cells may lead to suppressed expression of the “damage-associated molecular pattern” (DAMP) molecules necessary to alert the innate immune system to a “danger” incurred by the presence of aberrant cells [113]. Notably, even the “danger signals”, such as high-mobility group box 1 protein (HMGB1), can actually support cancer growth through stimulation of myeloid-derived suppressor cells (MDSCs) [114] or nurse-like cells [115] that create conditions favorable for cancer progression. Furthermore, tumor-associated specific antigens may assume forms similar to those expressed on normal cells and evade recognition as “non-self” by the immune system (reviewed in [116]).

Developing tumors create microenvironments that not only support neoplastic growth and metastasis, but also significantly reduce the effectiveness and corrupt the functions of both the innate and adaptive arms of anti-cancer immunity [10]. Among the immunosuppressive components of tumor microenvironments are various soluble factors such as IL-10, TGF-β, vascular endothelial growth factor (VEGF), prostaglandin E2 (PGE_2_), HMGB1, indoleamine-2,3-dioxygenase (IDO), as well as soluble forms of phosphatidylserine, Fas receptors, and MHC class I-related chain A proteins (reviewed in [117, 118]). Another recently recognized immunosuppressive mechanism involves the activation of the so-called immune checkpoints whose function is to prevent overstimulation of the immune system (reviewed in [119, 120]). The two most important immune checkpoint co-inhibitory molecules likely to play a role in induction and maintenance of the immunosuppressive state within tumors are members of the immunoglobulin gene superfamily, the cytotoxic T lymphocyte-associated antigen 4 (CTLA-4) whose expression on T helper cells suppresses the activity of cytolytic CD8^+^ T lymphocytes, and the programmed death 1 (PD-1; CD279) receptor primarily expressed on tumor-infiltrating lymphocytes (TILs) and monocytes which, upon combining with its respective ligands (PDL-1 and PDL-2), negatively regulates the anti-neoplastic function of T cells [121, 122]; in addition, the PD-1:PDL-1 interaction may promote the development and function of regulatory T (Treg) cells [123].

Active immunosuppression is also exerted by many non-specific and specific cellular effectors residing in or attracted to neoplastic tissue. Many different cells capable of inhibiting anti-cancer immunity and promoting cancer growth have now been identified. These include Treg lymphocytes [125, 126], MDSCs [127–130], macrophages [128, 131–133], natural killer T (NKT) [134–136], Th17 [137–139] and B lymphocytes [140–143], but also neutrophils [131, 144–147], dendritic cells (DCs) [148–151], mast cells (MCs) [152], and mesenchymal stem cells [153–155].

**Fig. 1:**
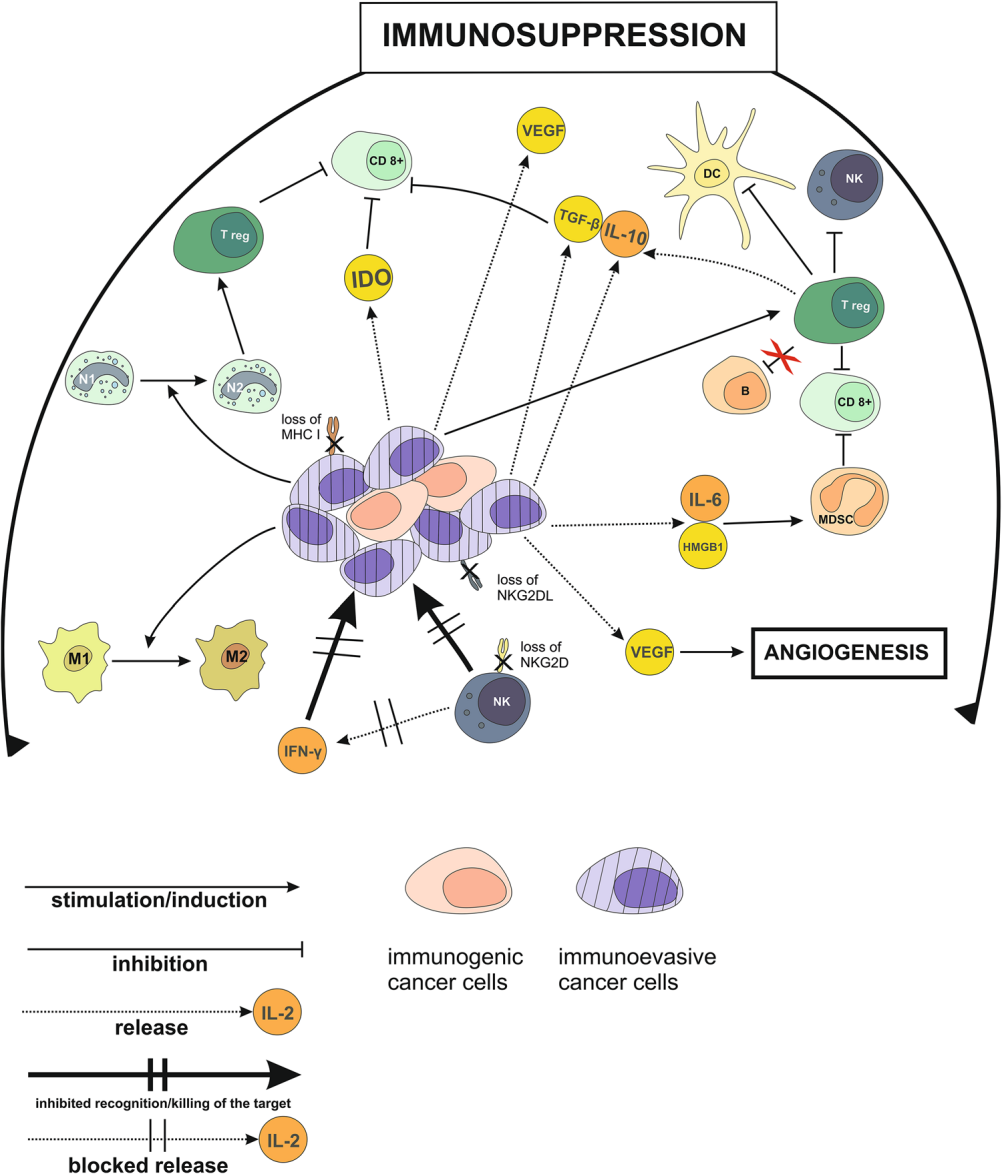
Tumor microenvironment during the late stages of cancer development: Immunosuppressive influences. *B* B lymphocytes, *CD8* ^*+*^ CD8^+^ T lymphocytes, *HMGB1* high-mobility group box 1 protein, *IDO* indoleamine-2,3-dioxygenase, *M1* phenotype 1 macrophages, *M2* phenotype 2 macrophages, *N1* phenotype 1 neutrophils, *N2* phenotype 2 neutrophils, *Treg* regulatory T lymphocytes, *NKG2DL* ligand for the natural killer group 2D receptor, *NKG2D* natural killer group 2D receptor, *VEGF* vascular endothelial growth factor

It has been finally well established that persistent activation of pro-inflammatory immunity facilitates cellular transformation and promotes tumor advancement. Unlike acute transient inflammatory responses which attract and activate elements of the innate immune system, chronic inflammation not only supports cancer progression, but also prevents the host from mounting effective immune defenses against it [129, 156–162]. An intermediate role in this process of the inflammation-driven type 2 immune response is played by MDSCs which are attracted to inflammatory sites and facilitate tumor growth [163, 164]. Chronic inflammation, as a powerful driver of carcinogenesis, is associated with aberrant signaling mediated by the nucleotide-binding oligomerization domain (NOD)-like receptors expressed on DCs, macrophages, and lymphocytes [165, 166]. Critical immunosuppressive mechanisms operating in the tumor microenvironment during the advanced stages of carcinogenesis are outlined in Fig. 1.

## Anti-neoplastic and immunomodulatory effects of LLR

### Overview

The development and progression of cancer in both humans and laboratory animals can be suppressed or prevented by exposures to LLR. The results of about 40 epidemiological studies published since 1987 have demonstrated decreased or unaltered cancer incidence or mortality rates in human populations exposed to LLR during medical diagnostic tests and therapy, in the course of professional activities, or as residents of geographical areas and homes with elevated levels of natural background radiation (evidence presented in Supplementary Table 1). Likewise, between 1996 and 2014, at least 27 reports were published from controlled experiments carried out in mice, rats, and dogs, as well as in cultured cells demonstrating that single, multiple, or chronic irradiations with LLR exert anti-neoplastic activities and markedly inhibit the growth and/or advancement of spontaneous or induced tumors (evidence presented in Supplementary Table 2). In general, the results of both epidemiological and experimental studies indicate or suggest that, in the case of short-term exposures at a high-dose rate, the upper threshold for the control of tumor growth is around 0.1 Gy [25, 61–63, 71, 167–169]. As evidenced by the results of experimental studies conducted in the in vivo and in vitro systems, one of the most important underlying mechanisms of such tumor-inhibitory effects is up-regulation of both the innate and adaptive immunity. Numerous reports published between 1988 and 2014 indicate that exposures to LLR are potent stimulators of various anti-neoplastic functions of the immune system, including inhibition of inflammation and/or up-regulation of anti-inflammatory cytokines (evidence presented in Supplementary Table 3 and reviewed in [74, 80, 94, 170, 171]).

### Specific studies demonstrating anti-tumor effects by LLR

There are also a number of reports dating back to early 1980s which demonstrate association of the LLR-induced up-regulation of anti-neoplastic immunity with inhibition of cancer development:

1. In 1982, Robert Anderson and collaborators [172] were among the first to report retardation of the growth of transplanted tumors in A/J mice following WBI with X-rays at doses ranging from 0.005 to 0.025 Gy immediately prior to s.c. inoculation of Sarcoma I cells. The evidence clearly suggested the involvement of “a very radiosensitive T cell with suppressor activity”.
2. In 1994, Kharazi et al. showed that chronic low-dose WBI with γ-rays (0.04 Gy per exposure, three times per week for 4 weeks) when combined with caloric restriction enhanced the regression of mammary tumors spontaneously developing in female C3H/He mice. These tumors were massively infiltrated with cytotoxic CD8^+^ T cells. Such tumor regression did not occur in mice subjected to caloric restriction alone [173].
3. As reported in 1999 by Hashimoto et al., a single WBI at 0.2 Gy of γ-rays of WKHA rats injected with hepatoma cells led to a significant reduction in the number of lung and lymph node metastases accompanied by the markedly stimulated influx of CD8^+^ lymphocytes into the spleen and the tumor site along with the enhanced expression of mRNAs for IFN-γ and TNF-α and down-regulation of mRNA for TGF-β; no mRNAs for IL-4, IL-6, and IL-10, the Th2-type cytokines that inhibit the anti-tumor Th1 responses, were detected in these tissues [69].
4. The studies by Yu et al. showed that a single exposure of male Kunming mice (a strain similar to C57BL/6 mice) to 0.075 Gy X-rays 6 h before implantation of S180 sarcoma cells significantly inhibited tumor growth accompanied by the influx of TILs as well as enhanced necrosis and down-regulation of the expression of receptors for VEGF in the neoplastic tissue [73, 74].
5. Continuous irradiation of C57BL/6 mice with γ-rays at 1.2 mGy/h for 258 days (up to 7.2 Gy total dose) did not induce thymic lymphomas, whereas the same total dose absorbed during four acute exposures to X-rays at 1.8 Gy resulted in the appearance of the lymphomas in 90% of these animals; in the continuously irradiated mice, the numbers of CD4^+^ T cells and antibody-producing B cells were significantly enhanced in the spleen [75].
6. Continuous exposure to γ-rays of the lymphoma-prone SJL/J mice at 100 mGy/y dose rate slightly prolonged life span of the animals and the effect was accompanied by the significant increase in the percentages of CD49^+^ NK cells and decreased percentages of CD4^+^ and CD8^+^ lymphocytes in the spleen [174]. When spleens of rats with a diethylnitrosamine-induced liver cancer were irradiated at 0.15 Gy from the 6 MeV β-beam accelerator at 100 mGy/min dose rate, the percentage of CD4^+^CD25^+^ Treg cells in the blood significantly decreased and the levels of Foxp3, IL-10, TGF-β, and CTLA-4 were down-regulated in the spleen and the tumor; these changes were accompanied by the suppressed tumor growth [175].
7. Experimental combinations of low-level WBI with the conventional (intermediate- or high-dose) local RT also yielded promising results: using murine tumor models of B16 melanoma and Lewis lung carcinoma, Liu and collaborators demonstrated that when fractionated local X-ray irradiations of the tumors at 2 Gy/fraction were several times substituted for WBI at 0.075 Gy, the cancer control (as judged by the reduced tumor mass and pulmonary metastases as well as by the increased survival of the hosts) was significantly improved compared to local RT alone; this effect was accompanied by up-regulation of the activities of the splenic NK and cytotoxic T lymphocytes which secreted elevated amounts of IFN-γ and TNF-α [77, 78].

## Our strategies showing anti-tumor effects by LLR

In a series of our own experiments carried out in the relatively radiosensitive BALB/c mice and the relatively radioresistant C57BL/6 mice, both single and multiple WBI with X-rays at total doses ranging from 0.05 to 0.2 Gy reproducibly suppressed development of the induced neoplastic colonies in the lungs. Since the mice were whole-body irradiated before inoculation of the syngeneic tumor cells, the obvious suggestion was that the low-level X-ray exposures stimulated systemic innate anti-neoplastic reactions. Although we were not able to directly estimate the activities of immune cells in the lungs, a significant stimulation of the cytotoxic activities of NK cells and LPS- and IFN-γ-stimulated macrophages obtained from the spleen and peritoneal cavity, respectively, was detected in the X-ray-exposed mice from both strains. Interestingly, no elevation of the activities of these cells was detected after their in vitro irradiation at the same doses of X-rays indicating that enhancing of the NK- and macrophage-mediated cytolytic functions by LLR depends on the presence of factors occurring in in vivo but not the in vitro conditions [81–90, 176–179].

## Clinical trials

The above-described epidemiological and experimental observations of anti-neoplastic and immunomodulatory effects of LLR exposures provide grounds for clinical trials with WBI or HBI of oncological patients [101, 180]. Even before the aforementioned evidence gained significance, a few LLR-based therapy trials had been performed. In 1965, Holder reported on positive therapeutic effects of the low-level total-body irradiation of patients with multiple myeloma [181]. In 1975, Kazem described curative effects of WBI (0.15 Gy of γ-rays daily for the first 5 days and thereafter at 0.1–0.15 Gy every other day or at longer intervals to the total doses of 2.0–2.65 Gy applied over 5–12 weeks) of patients with disseminated stage III lymphomas [182]. Likewise, Chaffey et al. obtained complete remissions in 32 out of 40 patients with advanced lymphocytic lymphoma after repeated WBI (0.15 Gy twice a week to a total dose of 1.5 Gy) as an initial and only primary therapy [102]. Very promising results of low-level total-body exposures to γ-rays of patients with non-Hodgkin’s lymphoma (NHL) were also reported by Qasim [183] and Choi et al. [103]. In one of the later trials, 24 out of 26 patients with stage IV low grade NHL were in complete remission after two courses of low-dose, total-body irradiation at 0.75 Gy given in five fractions; when the initially pathological lymph node areas of these patients were 1 month later treated with the conventional RT (total dose of 40 Gy applied in 20 fractions), the disease remitted in yet another patient [184]. Similarly, Safwat et al. who used low-level total-body exposures (0.1–0.25 Gy several times a week to the total dose of 1.5–2.0 Gy) obtained complete remissions in 11 out of 35 patients and 2-year progression-free survival in 12 patients with relapsed and/or chemo-resistant NHL; in 14 patients, a significant increase in the percentage of CD4^+^ T cells in the blood was noted [105]. In addition, as demonstrated by Sakamoto et al., low-dose HBI with X-rays (0.1–0.15 Gy two times a week for 5 weeks) combined with local RT (2 Gy five times a week for 6 weeks) resulted in the 5-year survival of 84% of patients with stage I and II NHL as compared to 65% survival of patients treated solely with local RT (the difference significant at *p* < 0.05); in these patients, percentages of peripheral blood CD4^+^ T helper lymphocytes were significantly elevated [100].

While more clinical trials employing WBI or HBI with LLR are needed, they are hampered by radiation safety regulations based on the linear, no threshold (LNT) model of the dose–effect relationship assuming that any absorbed dose of radiation causes a finite increase in cancer risk. There is a growing consensus that the LNT hypothesis lacks a solid experimental foundation and is based largely on ideology rather than science [25, 169, 185–193]. Hopefully, the many recent appeals from radiobiologists, physicians, and health physicists to various regulatory bodies and authorities to base the radiation protection system on scientific data indicating that there are quantitative and qualitative differences between the effects of low doses delivered at low dose rates and high doses delivered at high-dose rates [171, 187, 188, 190, 192, 194, 195] will lead to a revision of current radiation protection regulations, so that WBI with LLR can be tested in clinical trials.

## Suggested effects of LLR on cancer immunoediting process

**Fig. 2:**
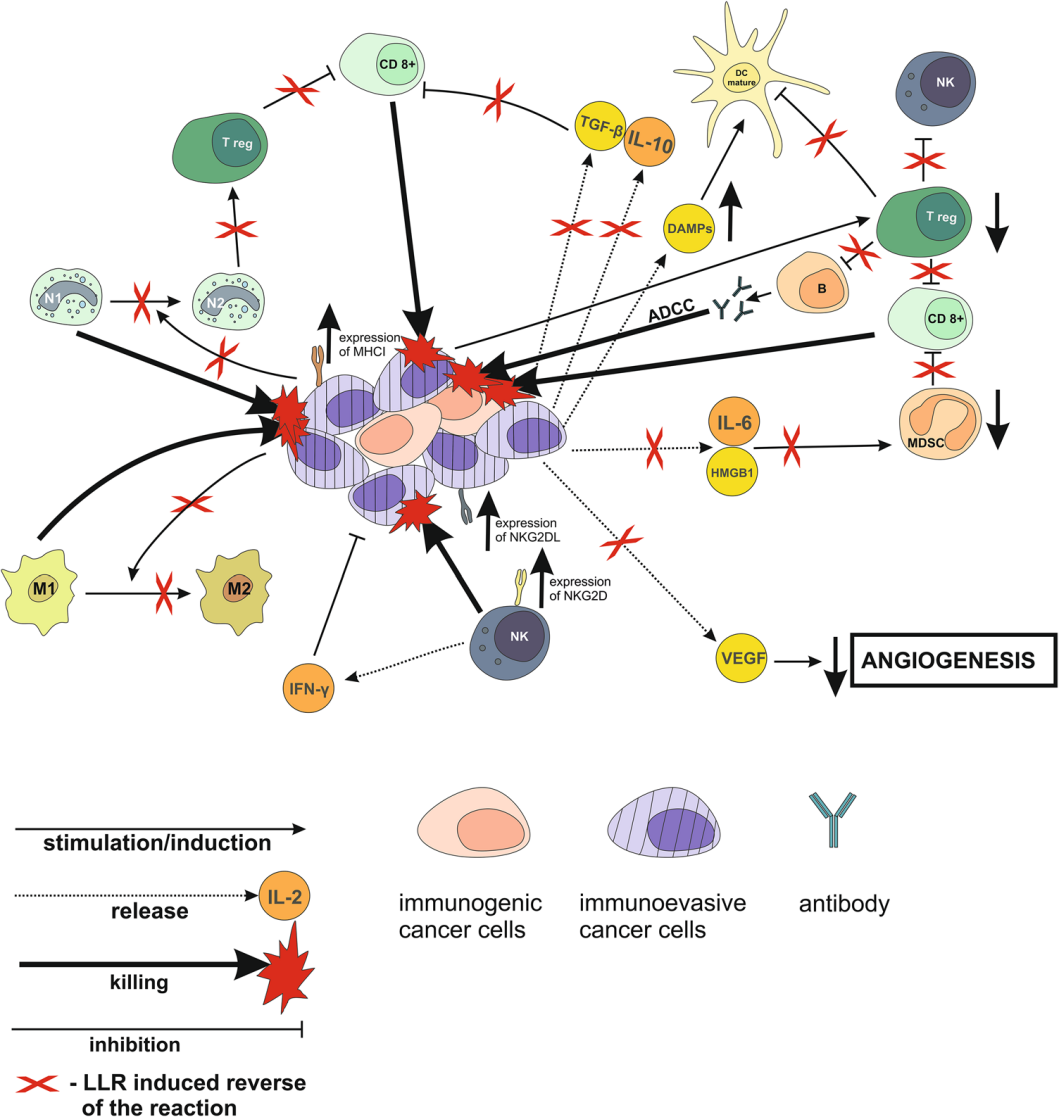
LLR-induced immune-related mechanisms mediating anti-neoplastic effects: Proposed framework. *ADCC* antibody-dependent cellular cytotoxicity, *B* B lymphocytes, *CD8* ^*+*^ CD8^+^ T lymphocytes, *DAMPs* damage-associated molecular pattern molecules, *HMGB1* high-mobility group box 1 protein, *M1* phenotype 1 macrophages, *M2* phenotype 2 macrophages, *N1* phenotype 1 neutrophils, *N2* phenotype 2 neutrophils, *Treg* regulatory T lymphocytes, *NKG2DL* ligand for the natural killer group 2D receptor, *NKG2D* natural killer group 2D receptor, *VEGF* vascular endothelial growth factor

As reviewed above, both acute and chronic exposures to LLR stimulate various anti-neoplastic immune reactions that are stifled or corrupted within the tumor microenvironment, especially during the later stages of carcinogenesis. Based on evidence indicating that tumor-inhibiting effects of LLR have been observed in both humans and experimental animals exposed in many different ways to single, multiple, and chronic irradiation with LLR, it may be argued that many, if not all, of the above-reviewed tumor-promoting immune mechanisms are likely to be blocked and/or reversed by such exposures (Fig. 2). Indeed, data indicating that LLR exposures may reverse the tumor-associated immune suppression has recently begun to emerge, even though many underlying LLR-induced mechanisms remain to be clarified. Based on the current evidence it may be postulated that, in addition to the direct activation of NK lymphocytes [83, 196, 197] and possibly other anti-tumor cytotoxic cells, LLR exposures enhance the “visibility” and/or susceptibility of cancer cells to immune assaults through stimulation of the expression by neoplastic and immune cells of molecules and ligands (e.g., CD2, B7, CD28, NKG2D) necessary for triggering of cytotoxic reactions [198–200] and/or turning on “danger signals” in the neoplastic tissue [201, 202]. Furthermore, low-level radiation exposures are likely to alleviate or reverse the tumor-associated immune degeneracy through elimination or inhibition of the multiple cells, cytokines, and other factors associated with immunosuppressive loops induced by the tumor [175, 203–207]. This could result in: (a) shifting of the immune response in favor of the anti-neoplastic phenotypes such as Th1 in the case of CD4^+^ T cells [97, 208], M1 in the case of macrophages [209, 210], and N1 in the case of neutrophils [211], (b) targeting the Treg-Th17 and Th17-DC interactions conducive to tumor regression [212–214], (c) activation of the Toll-like receptor-mediated signaling in phagocytes and antigen-presenting cells [215–217], (d) attenuation of the chronic inflammation pertinent to cancer initiation, promotion, and progression [94, 95, 170, 218, 219], and/or (e) down-regulation of the immune checkpoint molecules such as the CTLA-4, PD-1, and/or PD-L1 on T cells [198, 220–222]. Indeed, one of the recent reports indicates that hypofractionated γ-ray irradiation of tumors induced in C57BL/6 mice combined with blockade of the PD-1 checkpoint stimulated accumulation of TILs associated with complete eradication of very large neoplasms [222]. In addition, there are numerous non-immune mechanisms triggered by LLR that positively affect normal, but not malignant cells [29]. These include: (a) increased cell proliferation, (b) stimulation of anti-oxidant reactions associated with the reduction of tissue injury, (c) improved repair of the DNA damage, and (d) metabolic shift from oxidative phosphorylation to aerobic glycolysis resulting in increased radioresistance of healthy tissues. Such outcomes are of primary importance for the combination of the LLR-based immunotherapy with classic forms of cancer therapy (i.e., high-dose RT and chemotherapy) that are lethal to normal cells and tissues and promote the formation of reactive oxygen species and inflammation. It is expected that other LLR-triggered reactions and mechanisms will be detected providing additional grounds for the use of the truly low-level exposures to IR in the treatment of cancer and, possibly, other diseases.

## Conclusion and prospects

Cancer immunotherapy has matured from the application of several therapeutic agents, including tumor cell- and dendritic cell-based vaccines, anti-cytokine antibodies, checkpoint inhibitors, and genetically engineered T cells and stem cells, which collectively act to reverse immune suppression in the tumor environment and/or immune resistance of tumor cells (reviewed in [208]). There are also clinical trials combining such agents with local irradiation of tumors at moderate doses (i.e., >0.5–1.0 Gy per fraction) currently used in RT [16]. The recently acknowledged capacity of locally applied moderate or high (radiotherapeutic) doses of radiation to induce immunogenic death of cancer cells and local inflammatory reactions associated with stimulation of dendritic cells and enhancing the suppressed anti-cancer immunity has been employed as an adjuvant to improve the efficacy of existing immunotherapy protocols (reviewed in [11–19, 21]). However, such exposures can also cause persistent inflammation and multiple cell death in normal tissues, impede various immune and other physiological functions, and increase the risk of secondary primary cancers. In contrast, LLR exposures do not kill or impair and actually support functions of normal cells and tissues, selectively eliminate precancerous and transformed cells, attenuate rather than induce chronic inflammation, stimulate various anti-neoplastic reactions of the immune system, and are not associated with the development of secondary malignancies [21, 29, 94, 95, 170]. Finally, as indicated by the above-reviewed results of experimental and epidemiological studies as well as several clinical trials, WBI or HBI with LLR are not likely to induce any untoward side effects and can thus be used in treatment of patients with systemic or metastatic cancer.

It is, therefore, time to employ whole- or half-body exposures to LLR (alone or as an adjuvant to conventional therapeutics) to restore the efficacy of systemic anti-cancer functions of the immune system, the most potent guardian against neoplasia. This approach is expected to mediate improved clinical responses in cancer patients, as well as protect normal tissues from the well-known adverse effects associated with standard chemo- and radiotherapy used in contemporary cancer therapeutics.

## Electronic supplementary material

Below is the link to the electronic supplementary material.

Supplementary material 1 (PDF 505 KB)

## Abbreviations

ADCC: Antibody-dependent cellular cytotoxicity
DAMP: Damage-associated molecular pattern
Gy: Gray (the SI unit of absorbed dose defined as the absorption of 1 J of the radiation energy per 1 kg of matter)
HBI: Half-body irradiation
HMGB1: High-mobility group box 1 protein
IR: Ionizing radiation
LET: Linear energy transfer
LLR: Low-level radiation
LNT: Linear, no threshold
M1, M2: Macrophage phenotypes 1 and 2
MC: Mast cell
mGy: Milligray (0.001 Gy)
N1, N2: Neutrophil phenotypes 1 and 2
NHL: Non-Hodgkin’s lymphoma
NKG2DL: Ligand for the natural killer group 2D receptor
NKT: Natural killer T lymphocyte
NOD: Nucleotide-binding oligomerization domain
PGE_2_: Prostaglandin E2
RT: Radiotherapy
Th: Helper T lymphocyte
Treg: Regulatory T lymphocyte
VEGF: Vascular endothelial growth factor
WBI: Whole-body irradiation
