# Supplementary material for: Cancer immunotherapy: how low-level ionizing radiation can play a key role
Source: Cancer Immunol Immunother. 2017 Mar 30;66(7):819–32. doi: 10.1007/s00262-017-1993-z (PMC5489643; doi:10.1007/s00262-017-1993-z)
Supplement: Supplementary file 1 — Supplementary material 1 (PDF 505 KB) [file 262_2017_1993_MOESM1_ESM.pdf]

SUPPLEMENTARY TABLE 1

Cancer incidence and/or mortality in human populations (patients, workers, and residents) chronically exposed to low-level ionizing radiation.

Population	***Results of analyses***	***Reference***
Female radium dial painters (*workers*)	Bone sarcomas and carcinomas of the mastoid and paranasal sinuses diagnosed only in painters who had radiation doses to bone of at least 10 Gy	[30]
Residents of high natural radiation areas in Brasil, China, India, Iran, Japan, Poland	No excess or decreased cancer mortality or incidence rates compared to residents of low background radiation areas in these countries; in some studies a lower rate of cancer mortality correlated with enhanced reactivity of peripheral blood lymphocytes	[31-38]
Residents of dwellings with elevated levels of radioactive radon and its progeny – ecological studies	Inverse correlation between the concentration of radon in homes and lung cancer incidence and mortality among the residents	[40-43]
Female *patients* with tuberculosis exposed to multiple fluoroscopies	Breast cancer incidence in patients with radiation doses 0.05-0.3 Gy to the chest: 1/3 of background incidence	[44]
>7,800 *residents* of villages in the East Urals exposed to radioactive waste from the ‘Mayak’ nuclear reprocessing facility	Cancer incidence rates in groups of residents with effective doses up to 0.12 and 0.5 Sv 39% and 29% lower, respectively, than the expected baseline level in the area	[45]
American *radiologic technologists* (73% females) registered 1926-1980 and British	SMR for deaths from all cancers among radiological workers: 0.79-0.82 (American) and 0.71 (British)	[46-48]

male *radiologists* registered 1955-1979		
>14,000 *workers* at Sellafield plant of British Nuclear Fuels in Cumbria, England, exposed 1947-1975 to internalized plutonium and external low-LET radiation	Cancer mortality rate 5% lower than that of general population of England and Wales, and 3% lower than that of the general population of Cumbria	[49]
12,540 *workers* at the Capenhurst site of British Nuclear Fuels and its predecessors between 1946 and 1995	SMRs for all cancers = 88% and 97%, for radiation and non-radiation workers, respectively, and SIRs for all cancers = 82% and 88%, for radiation and non-radiation workers, respectively	[50]
Residents of the Apollo borough and the Parks township (in Armstrong and Westmoreland Counties, U.S.A) where uranium and plutonium processing facilities operated from 1965 to 1980	RRs of cancer death before start-up of the nuclear facilities (1950-1964), during the operations (1965-1980), and after plant closure (1980-1995) = 0.96, 0.95, and 0.98, respectively. RR of death from childhood leukemia = 1.02 before plant start-up, 0.81 during the operations, and 0.57 after closure.	[51]
>51,000 *workers* of the UK Atomic Energy Authority	All cancer mortality rates among radiation-exposed workers significantly lower compared to non-radiation workers	[52]
>38,000 nuclear shipyard *workers* exposed to radiation	Significantly reduced mortality rates due to cancer (SMR = 0.85), cardiovascular diseases, and respiratory diseases compared to the rates recorded in unexposed control	[53]

	workers	
Residents of counties near the Hanford nuclear facility in Richland, WA, U.S.A., releasing I-131 into the environment from 1944 to 1957	Overall cancer mortality rates slightly below those in similar counties in Washington State with minimal I-131 exposure; no excess mortality from thyroid cancer, female breast cancer, and leukaemia (including childhood leukaemia)	[54]
>7,200 *residents* of buildings in Taipei, Taiwan, chronically exposed to γ-radiation from the Co-60-contaminated steel used for construction	Significantly lower cancer incidence (SIR) for all cancers except leukaemia and for solid cancers (at 95% CI) and significantly lower SIR for all cancers (at 90% CI) compared to general population of Taipei	[55]
Residents of Montrose County, USA, living near uranium mining and milling facilities	In males: no excess mortality from cancers of the breast, kidney, liver, bone, leukemia, non-Hodgkin lymphoma or childhood cancer;<br>in females: decreased lung cancer mortality (RR= 0.85; 95% CI 0.67, 1.02) compared to non-exposed inhabitants of the county	[56]
Meta-analysis of >400,000 nuclear *workers* from 15 countries	Decreased risk of all cancers including leukemia compared to that recorded for non-exposed workers	[57,58]
Residents of homes in Worcester county, Massachusetts, with elevated radon levels – a case-control study	AORs for lung cancer risk among residents of homes where average radon level was 50, 75, and 150 Bq/m^3 were 0.53, 0.31 ($p<0.05$), and 0.47, respectively, compared to residents of homes with radon levels ≤25 Bq/m^3	[59,60]
Residents of the most contaminated areas of Belarus and Russia and	No increased rates of leukemias or solid tumors; elevated incidence of thyroid cancers only in children under 18 years of age in 1986	[61-64]

clean-up workers after the Chernobyl accident	who absorbed doses to the thyroid >0.1 Gy from incorporated I-131	
Childhood cancer *patients* treated with radiation therapy	Reduced incidence of second cancers per kg of tissue in regions of the body that had received radiation dose <0.2 Gy in comparison to non-irradiated regions	[65]
Hyperthyroid *patients* treated with I-131	In patients whose total body dose was 0.13-0.14 Sv the age-adjusted leukemia incidence was 11/100,000 patient-years vs. 14/100,000 patient-years in patients treated by surgery only	[27]
Residents of high impact states of USA where fallout from nuclear testing occurred	Lung cancer incidence in men and women from high-impact states significantly lower than that in residents of normal impact states	[66]
34,912 US male *radiologists* compared to 47,497 US male psychiatrists (both groups graduated from medical school in 1916-2006)	In the radiologists graduated after 1940: a) decreased RR of deaths from all malignant neoplasms and those of the CNS; b) insignificantly increased RR of deaths from total leukemia (but not in those graduated in 1960-1979); c) insignificantly increased RR of deaths from cancer of the respiratory organs (but not in those graduated in 1980-2006)	[223]
(i) 194,042 cases of breast cancer treated with beam radiation vs. 283,875 cases of breast cancer not treated with radiation; (ii)13,099 cases of rectosigmoid cancer treated with	(i) the small dose of scattered ovarian radiation (about 0.03 Gy) from beam radiation to the breast reduced the risk of ovarian cancer by 24% (ii) the beam radiation to the rectum that also reached the ovaries reduced the risk of ovarian cancer by 44% (iii) a significant inverse relationship detected between the incidence of ovarian cancer in	[224]

beam radiation vs. 33,305 cases of rectosigmoid cancer not treated with radiation	white women and radon background radiation and total background radiation.	

Legend: 95%/90% CI – confidence interval: the range of values in which 95/90% of the true means fall; AOR – adjusted odds ratio: the odds of exposure in the diseased group (cases) divided by the odds of exposure in the non-diseased group (controls); RR – relative risk (a.k.a. risk ratio): the ratio of the risk in the exposed group to the risk in the unexposed group; SIR - standardized incidence ratio: incidence in the studied cohort vs. incidence in the general population; SMR – standardized mortality ratio: mortality in the studied cohort vs. mortality in the general population; Sv – Sievert, an SI derived unit (1 Sv = 1 J/kg) of the equivalent and effective doses of ionizing radiation, for X- and gamma radiation 1 Sv roughly equals to 1 Gy.

SUPPLEMENTARY TABLE 2

Anti-neoplastic effects of LLR demonstrated in experimental systems.

Experimental system	***Irradiation***	***Effect***	***Reference***
Swiss mice	Five or ten WBI at 0.01 Gy γ-rays 24 h prior to the challenge dose of 2 or 3 Gy	Significant suppression of the development of thymic lymphoma compared to animals exposed to the challenge dose only	[67]
Quiescent mouse embryo C3H 10 T1/2 cells	Single irradiation with γ-rays at doses from 0.001 to 0.1 Gy	Three- to fourfold reduction of the risk of spontaneous neoplastic transformation after exposures at 0.001 up to 0.1 Gy; the protective effect seen only in irradiated cells allowed to incubate at 37oC before release from contact inhibition.	[225]
WKHA rats implanted into the right leg with KDH-8 hepatoma cells	WBI at 0.2 Gy γ-rays after implantation of the hepatoma cells	Significant suppression of metastases to the lungs and abdominal lymph nodes	[68,69]
CBA/H mice in which acute myeloid leukaemia (AML) was induced by chronic γ-ray irradiation to total dose of 1.0 Gy	Chronic irradiation at 0.1 Gy γ-rays 24 h prior to the chronic irradiation at 1.0 Gy	Significant increase in the latent period for the development of AML	[70]
Cancer-prone Trp53$^{+/-}$mice with reduced p53	Single exposures to γ-rays at 0.01 or 0.1 Gy, 0.5 mGy/min. dose rate	Significantly increased latency of lymphomas and spinal osteosarcomas after exposure at	[71]

function		0.01 Gy and significantly increased latency of lymphomas after exposure at 0.1 Gy	
HeLa x skin fibroblast hybrid cells	In vitro exposure to a range of doses of diagnostic energy (60 kVp) X-rays	At doses <0.01 Gy neoplastic transformation frequencies significantly less than those seen in non-irradiated cells	[72]
Kunming mice (similar to C57BL/6 mice) implanted into the right inguen with S180 sarcoma cells	WBI with X-rays at 0.075 Gy 6 h before sarcoma implantation	Significantly retarded tumor growth in the irradiated compared to non-irradiated control mice	[73,74]
C57BL/6 mice	Four acute WBI with X-rays at 1.8 Gy each (7.2 Gy total dose) or continuous irradiation for 258 or 450 days at 1.2 mGy/h (7.2 and 12.6 Gy total dose, respectively)	No thymic lymphomas in mice continuously exposed to X-rays vs. 90% of mice with lymphomas after four exposures at 1.8 Gy	[75]
AKR mice	WBI at 0.05 Gy three times a week or at 0.15 Gy two times a week from 11 weeks of age for 40 weeks.	Lymphoma incidence: 80.5% in sham-irradiated mice, 67.5% in mice irradiated with 0.05 Gy, and 48.6% in mice irradiated with 0.15 Gy; mean survival time significantly prolonged in all the irradiated mice.	[76]
Immature spermatocytes and spermatogonia of fruit fly	Exposure to X- rays at high (10 Gy) or low (0.2 Gy) total dose delivered at 0.05 Gy/min. dose rate	Compared to the sham-irradiated sperm cells significantly lower mutation frequency in the low does-exposed sperm cells and significantly increased mutation	[226]

Drosophila melanogaster		frequency in the high dose-exposed sperm cells	
C57BL/6 mice implanted with Lewis lung carcinoma (LLC) or melanoma (B16) cells	Conventional local RT with X-rays (each exposure at 2 Gy) interrupted with WBI at 0.075 or 0.1 Gy (LLR)	Significant inhibition of the transplanted tumor growth and reduction of pulmonary metastases in mice given combined RT and LLR treatment compared to those receiving local RT alone	[77,78]
Immature male germ cells of fruit fly *Drosophila melanogaster*	Irradiation with γ-rays at 22.4 mGy/h to a total dose of 0.5 mGy	Significantly decreased mutation frequency in the irradiated germ cells compared to the control, unexposed germ cells	[79]
Young beagle dogs	Inhalation of plutonium oxide ($^{238}PuO_2$, $^{239}PuO_2$) aerosols leading to absorbed lung doses from 0.08 to >77 Gy	Incidence of lung tumors significantly greater in control (zero dose) group than in the group with the lowest lung doses (0.08-0.22 Gy); no lung tumors in 16 dogs from the latter group	[80]
BALB/c mice i.v. injected with L1 sarcoma cells	Single WBI with X-rays at 0.1 or 0.2 Gy 2h before injection of the sarcoma cells	Significant inhibition of the development of sarcoma colonies in the lungs	[81-88]
BALB/c and C57BL/6 mice i.v. injected with L1 sarcoma and LLC cells, respectively	WBI with ten fractions of X-rays at 0.01, 0.02, or 0.1 Gy each before tumor cells injection	Markedly inhibited development of pulmonary tumor colonies	[88-90]
A/J mice i.p. injected with benzo(a)pyrene	0.6 Gy of γ-rays delivered in 6 bi-weekly fractions of 0.1 Gy starting one month after the injection of	Significant inhibition of the development of lung adenomas in the irradiated compared to non-irradiated mice	[91]

	benzo(a)pyrene		

SUPPLEMENTARY TABLE 3

LLR-induced modulation of immune functions in experimental systems

Experimental system	***Irradiation***	***Effect***	***Reference***
Wild-type C57BL/6 and autoimmune-prone C57BL/6 lpr/lpr mice	WBI with low-LET radiation at 0.01, 0.04 or 0.1 Gy/ day for 20 days	Increased proliferation of splenic T cells in both strains exposed at 0.01, 0.04 Gy/day dose rates, decreased proliferation of splenocytes from normal mice exposed at 0.1 Gy/day; shifts in proportions of T cells in the thymus and the spleen	[227,228]
C57BL/6 mice	WBI with γ-rays at 0.04 Gy	Increased production of IL-1 β and IFN-γ in the supernatants of co-cultures of splenic lymphocytes and peritoneal macrophages when the latter cells were obtained from the irradiated mice; enhanced production of IL-1β by the IFN-γ-treated macrophages obtained from the irradiated mice.	[229]
C57BL/6 and BALB/c mice	WBI with γ-rays at 0.04 Gy/day for 5 days	Significantly stimulated proliferation of splenic $CD8^+$ T cells in response to ConA in C57BL/6 mice; suppression of the same response in BALB/c mice	[230]
Kunming mice (similar to C57BL/6 mice)	Single WBI with X-rays at 0.075 Gy (low-level radiation, LLR) or 2.0 Gy (high-level radiation, HLR)	Up-regulation of CD28 and down-regulation of CTLA-4 on splenic T cells along with suppressed production of IL-10 (immunoenhancement) after LLR exposure; opposite effects (immunosuppression) after HLR exposure	[98,198]
Kunming mice	WBI with X-	Exposure to both low (0.05-0.075 Gy)	[215]

	rays at 0.05-2.0 Gy	and high (2 Gy) doses stimulated secretion by peritoneal macrophages of IL-12 and IL-18 accompanied by up-regulated expression of CD14 and TLR4-MD2 on the surface and of MyD88 in the cytoplasm of macrophages (activation of the Toll signalling pathway)	
BALB/c and C57BL/6 mice	Single WBI with X-rays at 0.1 or 0.2 Gy or ten whole-body irradiations at 0.01, 0.02, or 0.1 Gy each	Significantly enhanced cytotoxic activities of splenic NK cells and peritoneal macrophages and up-regulated secretion of NO, IL-1β, TNF-α, and IL-12 by macrophages and perforin, FasL, IL-2, and IFN-γ by splenocytes	[81-90, 176-179]
Co-culture of antigen-presenting cells (J774A.1) with T lymphocytes (EL-4)	Irradiation of J774A.1 cells with X-rays at 0.075 Gy (12.5 mGy/min. dose rate)	Stimulation by the irradiated J774A.1 cells of proliferation of nonirradiated T lymphocytes	[231]
Wild-type mouse strains (C57BL/6, BALB/c, C3H/He, DBA/1, DBA/2, CBA)	Chronic WBI with γ-rays at 1.2 mGy/h dose rate	Significant increase in $CD4^+$ and $CD8^+$ T cells, significant decrease in $CD40^+$ B cells, no $CD3^-CD4^+$ ("abnormal" immune cells) T cells in bone marrow, thymus, spleen, lymph nodes and/or peripheral blood	[232]
MRL-*lpr/lpr* mice with multiple diseases and shortened life span	Chronic WBI with γ-rays at 0.35 or 1.2 mGy/h dose rate	Significant prolongation of life span along with significant increase in $CD4^+CD8^+$ cells in the thymus and $CD8^+$ T cells in the spleen; significant decrease in $CD3^+CD45R/B220^+$ and $CD45R/B220^+CD40^+$ cells in the	[233]

		spleen	
C57BL/6 mice immunized with sheep red blood cells	Continuous WBI with X-rays at 1.2-mGy/h dose-rate till the end of life	Increased numbers of splenic $CD4^+$ T cells, $CD40^+$ B cells, and antibody-producing cells	[75]
C57BL/6 mice	WBI with 0.04 Gy γ-rays at 3.1 mGy/min. dose rate for 5 days at 24 h intervals (0.2 Gy total dose)	Enhanced NO secretion and phagocytosis by isolated peritoneal macrophages; in isolated splenic CD8+ T cells: (i) increased expression of CD69 antigen, (ii) enhanced proliferation in response to Con A, (iii) augmented mixed-leukocyte reaction and killing of target cells, (iv) suppressed secretion of IFN-γ	[234]
BALB/c and C57BL/6J mice	WBI with X-rays at 0.25 Gy	Increased numbers of plasma cells in spleens of the mice from both strains	[235]
Female C57BL/6 mice	WBI with X-rays at 0.2 Gy four times every other day for seven days	28 days post-exposure: increased numbers and functions of NK, T, and NKT lymphocytes in the liver, spleen and thymus	[236]
SJL/J female mice (spontaneously develop B-cell lymphoma)	Continuous WBI with γ-rays at 100 mGy/y dose rate	Slightly prolonged life span along with increased percentage of $CD49^+$ NK cells in the spleen and decreased percentages of $CD4^+$ and $CD8^+$ T cells in the lymph nodes and spleen	[174]
Murine dendritic cells (DCs) co-cultured with allogenic T cells	In vitro exposure to γ-rays at 0.02-1.0 Gy	DCs exposed to 0.05 Gy most vigorously stimulated proliferation of allogenic T cells, accompanied by increased production by DCs of IL-2, IL-12, and IFN-γ	[237]

C57BL/6 mice	WBI with proton radiation (as it occurs in the spaceflight environment) at total doses 0.01 or 0.1 Gy	Up-regulation of five to nine genes relevant to helper function of splenic $CD4^+$ T cells; no down-regulation of any of the 84 tested genes	[238]
C57BL/6 mice	WBI with γ-rays at total doses 0.01, 0.05, 0.1, and 0.5 Gy	In isolated splenocytes: (i) decreased apoptosis of $CD4^+$ T cells, Treg cells, $CD8^+$ cells, NK cells, DCs, and B cells; (ii) decreased expression of Th1-type cytokines (IL-2, IL-12, IFN-γ, LT-β and/or TNF-α) and Th2-type cytokines (IL-4, IL-6, IL-10); (iii) increased level of IL-5	[239]
Peripheral blood lymphocytes (PBLs) from healthy humans	In vitro irradiation with high-LET ^{12}C ions or low-LET X-rays at 0.05 Gy	Significant increase in cytotoxic activity of PBLs and their mRNA expression of IL-2, IFN-γ, and TNF-α; the effects more pronounced after exposure to ^{12}C ions	[240]
Peripheral blood lymphocytes (PBLs) from patients with alimentary tract and pancreatic cancer	As above	(i) Increased percentages of $CD3^+CD4^+$and $CD3^+$ $CD8^+$ T cells, (ii) increased expression of mRNAs for and secretion of IFN-γ and IL-2 by PBLs; the effects more pronounced after exposure to ^{12}C ions	[203-204]
C57BL/6 mice	WBI with γ-rays at 0.179 mGy/h to a total dose of 0.01 Gy (LLR) with or without	Increased production by splenic $CD4^+$ T cells of IL-2 and IL-4 and decreased production of TGF-β1 in the LLR + sSPE group compared to the sSPE only group	[241]

	subsequent exposure to simulated solar particle event (sSPE) protons at 1.7 Gy		
BALB/c mice	WBI with γ-rays at total dose of 0.2 Gy (15.44 mGy/h dose-rate)	(i) Stimulated differentiation of bone marrow cells into DCs expressing CD80 and CD86 and MHC class I and II molecules (on immature and mature DCs), (ii) increased serum levels of G-CSF and IL-10.	[242]
C57BL/6 mice developing streptozotocin-induced diabetes	WBI with X-rays at 0.025 Gy once every 2 days for 2, 4, 8, 12 and 16 weeks	Significant reduction of diabetes-induced cardiac inflammation accompanied by down-regulation of cardiac levels of IL-18, TNF-α, MCP-1, and PAI-1	[170]
C57BL/6 mice developing streptozotocin-induced diabetes	WBI with X-rays at 0.05 or 0.075 Gy for 4 weeks with high-fat diet treatment	Significant attenuation of inflammation and oxidative stress in the diabetic kidneys	[95]
Rat basophilic leukemia cells (RBL-2H3), a model system for studying mast cells	In vitro exposure to γ-rays at 0.01 Gy	(i) suppressed release of histamine, β-hexosaminidase, IL-4, and TNF-α from IgE-sensitized RBL-2H3 cells, (ii) inhibition of phosphorylation of signaling molecules following stimulation of the high-affinity IgE receptor FcεRI	[243]
Purified NK cells from spleens of C57BL/6 mice	In vitro exposure to γ-rays at 4.2	Significant stimulation of cytotoxic function of NK cells against the MHC class I-negative RMA/S target cells	[196]

	mGy/h to a total dose of 0.2 Gy		
Isolated human primary monocytes	In vitro irradiation with X rays at 0.05 and 0.1 Gy	Activation of pro-survival and pro-inflammatory responses: up-regulation of TLR signaling molecules (HMGB1, TLR4, TLR9, MyD88 and IRAK1), and the NF-κB and MAPK pathways	[244]
C57BL/6 mice	WBI with γ-rays at 30 mGy/h to a total dose of 0.01 Gy (LLR) with or without 2 Gy proton (P; 1 Gy/min.) or γ-ray (γ; 0.9 Gy/ min.) irradiation	(i) High percentage of $CD4^+$ $CD25^+$ $Foxp3^+$ Treg cells in spleens from P vs. P + LLR and LLR + P groups vs. control (0 Gy) group; (ii) in spleen supernatants: high IL-2 level in LLR and LLR + P groups vs. 0 Gy group; high IL-10 level in LLR + γ groups vs. 0 Gy group; (iii) low TGF-β1 level in the blood from LLR + P vs. LLR + γ groups; (iv) activated cJun N-terminal kinase (JNK) in $CD4^+$ T cells from LLR + P vs. LLR + γ group	[245]
NK cells isolated from peripheral blood of healthy human donors	In vitro irradiation with X-rays at 12.5 mGy/min to a total dose of 0.075 Gy	Expansion and stimulation of the cytotoxic activity of NK cells accompanied by significantly increased levels of IFN-γ and TNF-α in supernatants of cultured NK cells	[197]

Additional, supplementary references:

223. Berrington de González A, Ntowe E, Kitahara CM, Gilbert E, Miller DL, Kleinerman RA et al (2016) Long-term mortality in 43 763 U.S. radiologists compared with 64 990 U.S. psychiatrists. Radiology 281:847-857. doi: 10.1148/radiol.2016152472
224. Lehrer S, Green S, Rosenzweig KE (2016) Reduced ovarian cancer incidence in women exposed to low dose ionizing background radiation or radiation to the ovaries after treatment for breast cancer or rectosigmoid cancer. Asian Pac J Cancer Prev 17:2597-2600. doi:10.7314/APJCP.2016.17.6.2597
225. Azzam EI, de Toledo SM, Raaphorst GP, Mitchel RE (1996) Low-dose ionizing radiation decreases the frequency of neoplastic transformation to a level below the spontaneous rate in C3H 10T1/2 cells. Radiat Res 146:369-373. doi: 10.2307/3579298
226. Koana T, Okada MO, Ogura K, Tsujimura H, Sakai K (2007) Reduction of background mutations by low-dose X irradiation of Drosophila spermatocytes at a low dose rate. Radiat Res 167:217-221. doi: 10.1667/RR0705.1
227. James SJ, Makinodan T (1988) T cell potentiation in normal and autoimmune-prone mice after extended exposure to low doses of ionizing radiation and/or caloric restriction. Int J Radiat Biol Relat Stud Phys Chem Med 53:137-152. doi: 10.1080/09553008814550491
228. James SJ, Enger SM, Peterson WJ, Makinodan T (1990) Immune potentiation after fractionated exposure to very low doses of ionizing radiation and/or caloric restriction in autoimmune-prone and normal C57Bl/6 mice. Clin Immunol Immunopathol 55:427-437. doi: 10.1016/0090-1229(90)90129-E
229. Ibuki Y, Goto R (1999) Contribution of inflammatory cytokine release to activation of resident peritoneal macrophages after in vivo low-dose gamma-irradiation. J Radiat Res 40:253-62. doi: 10.1269/jrr.40.253
230. Shankar B, Premachandran S, Bharambe SD, Sundaresan P, Sainis KB (1999) Modification of immune response by low dose ionizing radiation: role of apoptosis. Immunol Lett 68:237-245. doi: 10.1016/S0165-2478(99)00074-7
231. Liu SZ, Jin SZ, Liu XD (2004) Radiation-induced bystander effect in immune response. Biomed Environ Sci 17:40-46
232. Ina Y, Sakai K (2005) Activation of immunological network by chronic low-dose-rate irradiation in wild-type mouse strains: analysis of immune cell populations and surface molecules. Int J Radiat Biol 81:721-729. doi: 10.1080/09553000500519808

233. Ina Y, Sakai K (2005) Further study of prolongation of life span associated with immunological modification by chronic low-dose-rate irradiation in MRL-lpr/lpr mice: effects of whole-life irradiation. Radiat Res 163:418-423. doi: 10.1667/RR3316
234. Pandey R, Shankar BS, Sharma D, Sainis KB (2005) Low dose radiation induced immunomodulation: effect on macrophages and CD8+ T cells. Int J Radiat Biol 81:801-812. doi:10.1080/09553000500531886
235. Kataoka T, Mizuguchi Y, Notohara K, Taguchi T, Yamaoka K (2006) Histological changes in spleens of radio-sensitive and radio-resistant mice exposed to low-dose X-ray irradiation. Physiol Chem Phys Med NMR 38:21-29
236. Ren H, Shen J, Tomiyama-Miyaji C, Watanabe M, Kainuma E, Inoue M et al (2006) Augmentation of innate immunity by low-dose irradiation. Cell Immunol 244:50-56. doi: 10.1016/j.bbadis.2010.11.010
237. Shigematsu A, Adachi Y, Koike-Kiriyama N, Suzuki Y, Iwasaki M, Koike Yet al (2007) Effects of low-dose irradiation on enhancement of immunity by dendritic cells. J Radiat Res 48:51-55. doi: 10.1269/jrr.06048
238. Gridley DS, Pecaut MJ, Rizvi A, Coutrakon GB, Luo-Owen X, Makinde AY et al (2009) Low-dose, low-dose-rate proton radiation modulates CD4(+) T cell gene expression. Int J Radiat Biol 85:250-261. doi: 10.1080/09553000902748609
239. Bogdándi EN, Balogh A, Felgyinszki N, Szatmári T, Persa E, Hildebrandt G et al (2010) Effects of low-dose radiation on the immune system of mice after total-body irradiation. Radiat Res 174:480-489. doi: 10.1667/RR2160.1
240. Chen Y, Li Y, Zhang H, Xie Y, Chen X, Ren J et al (2010) Early effects of low dose C ion or X-ray irradiation on human peripheral blood lymphocytes. Adv Space Res 45:832-838. doi: 10.1016/j.asr.2009.09.024
241. Rizvi A, Pecaut MJ, Slater JM, Subramaniam S, Gridley DS (2011) Low-dose γ-rays modify CD4(+) T cell signalling response to simulated solar particle event protons in a mouse model. Int J Radiat Biol 87:24-35. doi: 10.3109/09553002.2010.518206
242. Chun SH, Park GY, Han YK, Kim SD, Kim JS, Lee CG et al (2012) Effect of low dose radiation on differentiation of bone marrow cells into dendritic cells. Dose Response 11:374-384. doi: 10.2203/dose-response.12-041.Lee
243. Joo HM, Nam SY, Yang KH, Kim CS, Jin YW, Kim JY (2012) The effects of low-dose ionizing radiation in the activated rat basophilic leukemia (RBL-2H3) mast cells. J Biol Chem 287:27789-27795. doi: 10.1074/jbc.M112.378497
244. El-Saghire H, Michaux A, Thierens H, Baatout S (2013) Low doses of ionizing

radiation induce immune-stimulatory responses in isolated human primary monocytes. Int J Mol Med 32:1407-1414. doi: 10.3892/ijmm.2013.1514

245. Gridley DS, Rizvi A, Makinde AY, Luo-Owen X, Mao XW, Tian J et al (2013) Space-relevant radiation modifies cytokine profiles, signaling proteins and Foxp3+ T cells. Int J Radiat Biol 89:26-35. doi: 10.3109/09553002.2012.715792
